# Qualitative and quantitative evaluation of the usability of transport ventilators using eye tracking

**DOI:** 10.1038/s41598-025-34154-5

**Published:** 2026-01-08

**Authors:** Axel Schmutz, Theresa Schwarz, Stefan Schumann, Johannes Spaeth

**Affiliations:** 1 Department of Anesthesiology and Critical Care, Medical Center, University of Freiburg, Hugstetter Strasse 55, 79106 Freiburg, Germany; 2 Faculty of Medicine, University of Freiburg, Freiburg, Germany

**Keywords:** Critical care, Patient safety, Medical device design, Mechanical ventilator, Pre-hospital medicine, Portable ventilator, Transport ventilator, Eye tracking, Heatmaps, Hot spots, Biomedical engineering, Respiratory tract diseases, Respiratory distress syndrome, Hypoxia

## Abstract

Deficiencies in the usability of transport ventilators may lead to operating errors and patient harm. Intuitively operable devices and optimized ergonomics can reduce cognitive workload and critical events. In order to identify shortcomings of specific devices we compared the user interfaces of four modern transport ventilators. We hypothesized that the task-specific usability of user interfaces differs between transport ventilators. Therefore, we investigated the usability of modern transport ventilators from four manufacturers by means of subjective and objective usability measures, including eye-tracking analysis in 20 typical subsequent tasks. Thirty-four anesthesia residents without extensive experience in the use of transport ventilation were asked to perform a series of tasks on each of four transport ventilators of different manufacturers (Hamilton-T1, Medumat Transport, Dräger Oxylog 3000 plus and Monnal T60). Number of assistances and task failure rates were counted, and task completion times were measured. Further the systems’ usability was assessed via questionnaires and rating scales. In addition, eye-tracking was used to analyze gaze direction, time to first fixation and heatmaps superimposed to snapshots. Data are presented as means ± SD and compared by Chi Square tests or two-way ANOVA where appropriate. Task failure rate was highest with the Monnal T60 (10.9%), followed by Hamilton-T1 (8.6%), Oxylog 3000 plus (4.2%) and Medumat Transport (3.0%). Cumulative task completion times differed between the devices. To render all tasks took longest with the Monnal T60 (368 ± 228 s), followed by the Hamilton-T1 (294 ± 136 s), the Oxylog 3000 plus (214 ± 116 s) and the Medumat Transport (204 ± 111 s). System usability scores were comparable between the Oxylog 3000 plus, the Hamilton-T1 and the Medumat Transport, but significantly lower for the Monnal T60. Eye movement analysis showed that, depending on the device and task, some functions were recognized quickly and tasks were solved immediately. Heatmaps showed marked differences in operators´ visual focuses. The systems under test revealed relevant task and design related differences in their usability. The comparison of the four devices highlights the importance of intuitive user guidance to reduce potential sources of error.

**Supplementary Information:**

The online version contains supplementary material available at 10.1038/s41598-025-34154-5.

## Background

Transport ventilators are designed to ventilate critically ill patients in unstable working environments under time-sensitive conditions, for example, during intra-hospital transport. Evidence from comparatively complex and demanding circumstances shows that such conditions increase the risk of device-related incidents caused by human factor issues ^1–3^. Accordingly, setting of appropriate ventilation parameters and interpreting respiratory variables requires a high level of user awareness. Intuitively operable machines can reduce the user’s cognitive workload and consequently the risk of critical incidents ^4^. For this reason, medical device authorities increasingly call for user centered design ^5^. Thereby, the design of a device’s user interface is of crucial importance ^6^.

In our study we aimed to investigate the user interfaces of four representative transport ventilators from different manufacturers. For that purpose, anesthesia residents were asked to perform a series of tasks on each device during simulated ventilation. We hypothesized that the task-specific usability depends on the design of the respective item of the user interface. Therefore, the success of task performance, the number of requested assistances and task completion times were evaluated. By measuring an individual’s fastest response to a stimulus, eye-tracking provides detailed insights into behavioral task processing. In the context with task solving, this allows for evaluating perceptual speed, distraction effects, and assessment of cognitive load relative to the user guidance offered. Therefore, the operators’ visual focuses were measured using a mobile eye-tracking device and analyzed, additionally. Finally, the user experience was evaluated via a standardized usability questionnaire.

## Material and methods

### Transportable ventilators under test

Four sophisticated transport ventilators, representing the most commonly used devices in the European market, that offer multiple ventilation modes and visualization of the pressure/volume curves from different manufacturers were included in the study: Monnal T60 (Software Version v2.1.3, Air Liquide Medical Systems S.A., Antony Cedex, France); Oxylog 3000 plus (Software Version 01.04, Drägerwerk AG & Co. KGaA, Lübeck, Germany); Hamilton-T1 (Software Version 2.2.0, Hamilton Medical AG, Bonaduz, Switzerland); Medumat Transport (Software Version 6.5, WEINMANN Emergency Medical Technology GmbH + Co. KG, Hamburg, Germany). Following the authors’ request, all devices were provided by the manufacturers without any incentives being given.

The differences between the user interfaces of the devices examined are as follows: Two of the devices are operated via a touchscreen and an additional navigation knob (Monnal T60), and additional function keys (HAMILTON-T1). In contrast, the other two devices use a display without touch functionality. These devices use function keys, a navigation knob (Oxylog 3000 plus), and additional context-sensitive keys (MEDUMAT Transport) to access settings and navigate the menu.

### Operators

To include operators with a sufficient understanding of ventilation therapy but without extensive experience in the use of transportable ventilators, anesthesia residents were assessed for eligibility at our university medical center. Postgraduate anesthesia training in Germany typically takes five years, but we aimed at including residents before they had started their intensive care and emergency medicine training in the final year of residency. Participation was refused after intake of sedative drugs within 12 h and if reaction times, measured in three repetitions using an Android App (Reaction Time v1.5.1 Plus, Ewefo), exceeded 350 ms. The study was approved by the local ethics committee (Ethics Committee of the University of Freiburg, Approval No. EK 56/17) and written informed consent was obtained from all volunteers before they had participated. All experiments were performed in accordance with relevant guidelines and regulations.

### Task definition

In order to set reliable tasks, representing a typical procedure of using a transport ventilator, these were defined using the following procedure. First, tasks were derived from earlier studies with a comparable protocol evaluating anaesthesia ventilators ^7^. In a second step, this sample was subject to a process of selection and rewording. Therefore, senior consultants specialized in anesthesia, emergency medicine and intensive care medicine were consulted. The 20 tasks which have been finally included are listed in Table 1. Tasks were always presented in the same order, again with the intention to represent the tasks in the context of a typical use, and further to prevent from conflicting task orders. In tasks including variables to be set, values were varied between ventilators to avoid rendering the task ahead of time.

**Table 1:** Formulation of the tasks.

1	Connect the ventilation hose system to the device
2	Turn on the device
3	Set volume-controlled ventilation mode
4	Set tidal volume to … milliliter
5	Set ventilation frequency to … per minute
6	Set positive end-expiratory pressure (PEEP) to …
	Start ventilation (ventilation started automatically in certain devices)
7	Set peak pressure to …
8	Set inspiratory O_2_ to 100 percent oxygen
9	Set inspiratory to expiratory ratio to…
10	Read out the maximum inspiratory pressure
11	Read out the expiratory minute volume
12	Set pressure support ventilation mode
13	Open the menu to change the alarm limits
14	Set the lower limit for respiratory minute volume to … liter per minute
15	Set the alarm volume to …
16	Activate pressure controlled ventilation mode
17	Read out the end-tidal carbon dioxide concentration
18	Read out the expiratory tidal volume
19	Read out the battery state of charge
20	Turn off the device

### Experimental setup

The study was conducted in a calm test environment. Each device was placed in a separate cabinet. A white background was used to avoid user distraction. The operators’ gaze was measured by means of a mobile eye-tracker glasses system, during the tasks. Therefore, ambient light was slightly dimmed, to achieve appropriate tradeoff between screen and eye-tracking ambient conditions. The ventilators were presented at an individual adjustable height, appropriate for task completion in a sitting position. This way, an orthogonal perspective towards the screen could be approximated. For certain tasks, the ventilators were connected to a test lung (Test Lung 1.5 L, Dräger, Lübeck, Germany), using ventilation tubing appropriate for the respective device. Before each experimental session, a functional check was run according to the manufacturers’ instructions. Before starting the measurements, ventilators were covered with a blanket in order to avoid visual impressions not assessed by measurements.

### Study protocol

Participants were scheduled for individual appointments. Age, height, professional experience, ventilator experience and reaction times were recorded. Afterwards, eye-tracking glasses (Tobii Pro Glasses 2, Tobii AB, Stockholm, Sweden) were fitted and calibrated following manufacturers’ instructions. Ventilators were presented in randomized order. The respective sequence was disclosed just before the session was started. Once the operator had sat down in front of the device, the eye-tracking recordings were started and the device was uncovered, allowing the operator to visually inspect it for one minute without touching the device or exploring the menu options. These eye-tracking measurements without task will be referred to as *hot-spot measurements* in the following.

Operators were instructed to render the following tasks quickly but diligently. Thereafter, tasks were verbally stated via word for word readout, from the investigator standing laterally to the sitting operator. The operator then started to render the task at the operator’s discretion. While rendering a task, operators were able to claim up to three assistances from the investigator. If the operator faced obstacles but did not ask for assistance, assistance was given after 60, 120 and 180 s. Tasks were rated successfully executed at the investigators decision. However, with the intention to prevent from excessive operator frustration or fatigue due to very long experimentation time, cutoffs were determined, as follows. If more than three assistances were requested or if the operating time for a task exceeded 240 s, the task was rated `failed´. The durations required to render the task were determined retrospectively from the eye-tracking video recordings. Task completion time (TCT) was defined as the time from the first word of the verbalized task until the last contact with the device or the end of the given answer.

After having completed all tasks at a respective device, operators were asked to rate their experienced stress by setting a mark on a continuous scale between zero (indicating no stress) and 100 (indicating maximum perceived stress). Subsequently, they were asked to rate their immediate experience utilizing the System Usability Scale (SUS) ^8^, a standardized questionnaire for estimation of global acceptance of a systems’ usability. The SUS scores range from 0 to 100, with larger scores indicating better usability ^9^. Scores of the individual items are however not meaningful on their own.

Two additional questions were included about whether the feedback from the device was understandable and whether the layouts of the displays were clear, with agreement rated on a 5-point Likert scale.

### Eye-tracking analysis

The gaze points were manually mapped on a snapshot image showing a strict two-dimensional view of the respective ventilator´s interface ( [1.34] Tobii Pro Glasses Analyser, www.tobii.com/products/software/behavior-research-software/tobii-pro-lab). Manual mapping was always performed by the same investigator (TS). From the gaze points’ regional and temporal distribution, fixations were classified if the velocity of the eye movement was below a threshold of 30°/s.

The aim of the eye-tracking analysis was to elucidate the cause of disturbance for tasks with significant differences in TCT. For automated analysis of the eye-tracking data, areas of interest (AOI) were set to indicate the displayed screen area or button in question at the mapped snapshots. With this, Time to First Fixation (TFF) within an AOI was calculated indicating the time that had passed until the operators looked first into the respective AOI. If a task required two activation steps or the respective AOI lay within a submenu, two snapshots were used to map the successive operating steps and times were added up automatically.

Spatial representation of eye-movement data were quantitatively illustrated generating heatmaps superimposed to snapshots. Therefore, the peripheral vision around a fixation point was accomplished using an approximation to the Gaussian curve with a default radius of 50 pixels reflecting the human visual field. At the heatmaps, the operators’ mean fixation counts appear colored, in which warm colors (red) represent a high number of fixations, followed by yellow and green.

### Statistics

The required sample size for statistical analysis could not be determined from a power analysis or existing literature. Therefore, sample size was estimated based on the expected ratings of the System Usability Scale (Score range 0–100). In a preceding study with comparable protocol ^7^, the systems demonstrated a mean scale figure of 59 with a standard deviation [SD] of 19. We considered a difference of 13 and an alpha error of 0.05 to be relevant. Thus a minimum sample size of 27 operators was necessary to reach 0.8 test power. We, however, aimed at including more operators for the following reasons: first, it is recommended to increase the number of test persons for evaluation of the usability of medical equipment with safety-relevant intended purpose; second, based on our experience from a previous trial, a possible higher dropout rate due to artefacts in the eye-tracking signal was taken into account. Finally, 34 participants were recruited for the experiments. This figure lies within the scope of usability testing studies ^10,11^.

Data are presented as means and SD if not indicated otherwise. The number of failed tasks and first assistances for each task between ventilators were compared by Chi Square tests, TCT and TFF were compared by Two-way ANOVA for each task between the ventilators. Assuming sufficient continuity and thus sufficient parametricity, SUS scores, the experienced level of stress and questions about the user-interface feedback and clarity were compared by one-way repeated measures ANOVA, followed by Tukey–Kramer tests if applicable (all statistics performed with [9.5.1] GraphPad PRISM, GraphPad Software Inc., La Jolla, California, USA; https://www.graphpad.com). A p-value lower than 0.05 was considered significant.

## Results

Data of all 34 operators (mean age 30 yrs (range 25–45 yrs; 13 female) could be included in the analysis. The mean duration of postgraduate anesthesia training was 21 months (range 1 to 48 months). Since it is the standard device for transport ventilation in our department, 24 operators had experience with the Oxylog 3000 plus ranging from use on two occasions in total to sporadic use within recent years. One test person had sporadically used the Medumat Transport. The operators’ average reaction time was 256 ms (range 213 to 334 ms) before beginning of the experiments.

### Task failure rates

**Fig. 1:**
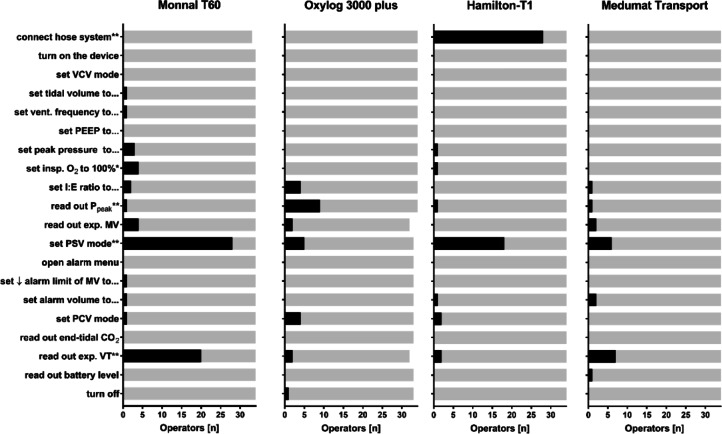
Task failure rate for all included ventilators. Tasks in the order of study protocol (vertically top down). Bars indicate total count. * *p* < 0.05, ** *p* < 0.001 regarding at least one comparison of error counts of the same task between different devices, calculated by Chi Square Test.

Task failure rates differed depending on the device in question (*p* < 0.0001; Fig. 1). The highest failure rate was found with the Monnal T60 (10.9%), followed by Hamilton-T1 (8.6%), Oxylog 3000 plus (4.2%) and Medumat Transport (3.0%). Thereby, with the Hamilton-T1 52% of the task failures concerned the connection of the hose system (Task 1). With the Monnal T60 42% of the task failures happened during activation of pressure support ventilation mode (Task 12). Most task failures occurred because operators were unable to approach the respective task, despite assistance, but not because they solved the task incorrectly.

### Assistances

The cumulative number of given assistances per device (not including failed tasks) was highest with the Monnal T60 (55 assistances), followed by Hamilton-T1 (44 assistances), Oxylog 3000 plus (12 assistances) and Medumat Transport (6 assistances; *p* < 0.0001). Most assistances per task were needed with the tasks `Setting peak pressure to…´ (Task 7; 21 assistances in the Hamilton-T1; 10 assistances in the Monnal T60) and `Open the menu to change the alarm limits´ (Task 13; 18 assistances in the Monnal T60).

### Task completion times

**Fig. 2:**
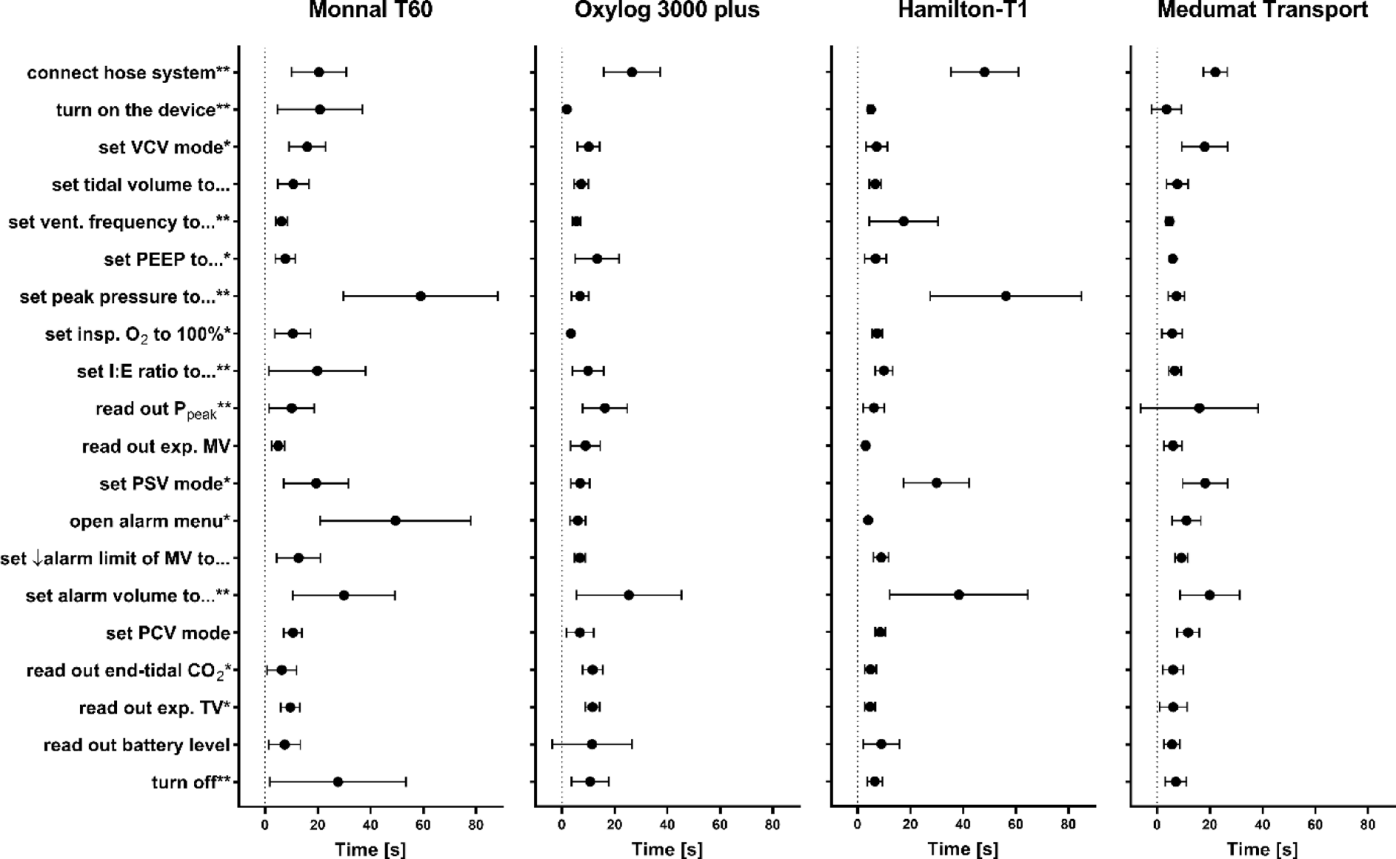
Task completion times for all included ventilators in the order in which tasks were tested (vertically top down). Tasks rated failed were excluded from the analysis. Bars indicate means ± standard deviation. * *p* < 0.05, **p < 0.001 regarding at least one comparison of the same task between different devices, calculated by two-way ANOVA.

Cumulative TCTs (not including failed tasks) differed between the tested devices. To render all tasks took longest with the Monnal T60 (368 ± 228 s), followed by the Hamilton-T1 (294 ± 136 s), the Oxylog 3000 plus (214 ± 116 s) and the Medumat Transport (204 ± 111 s). Specific TCT differed between the devices (Fig. 2; *p* < 0.0001).

### Usability score results.

**Fig. 3:**
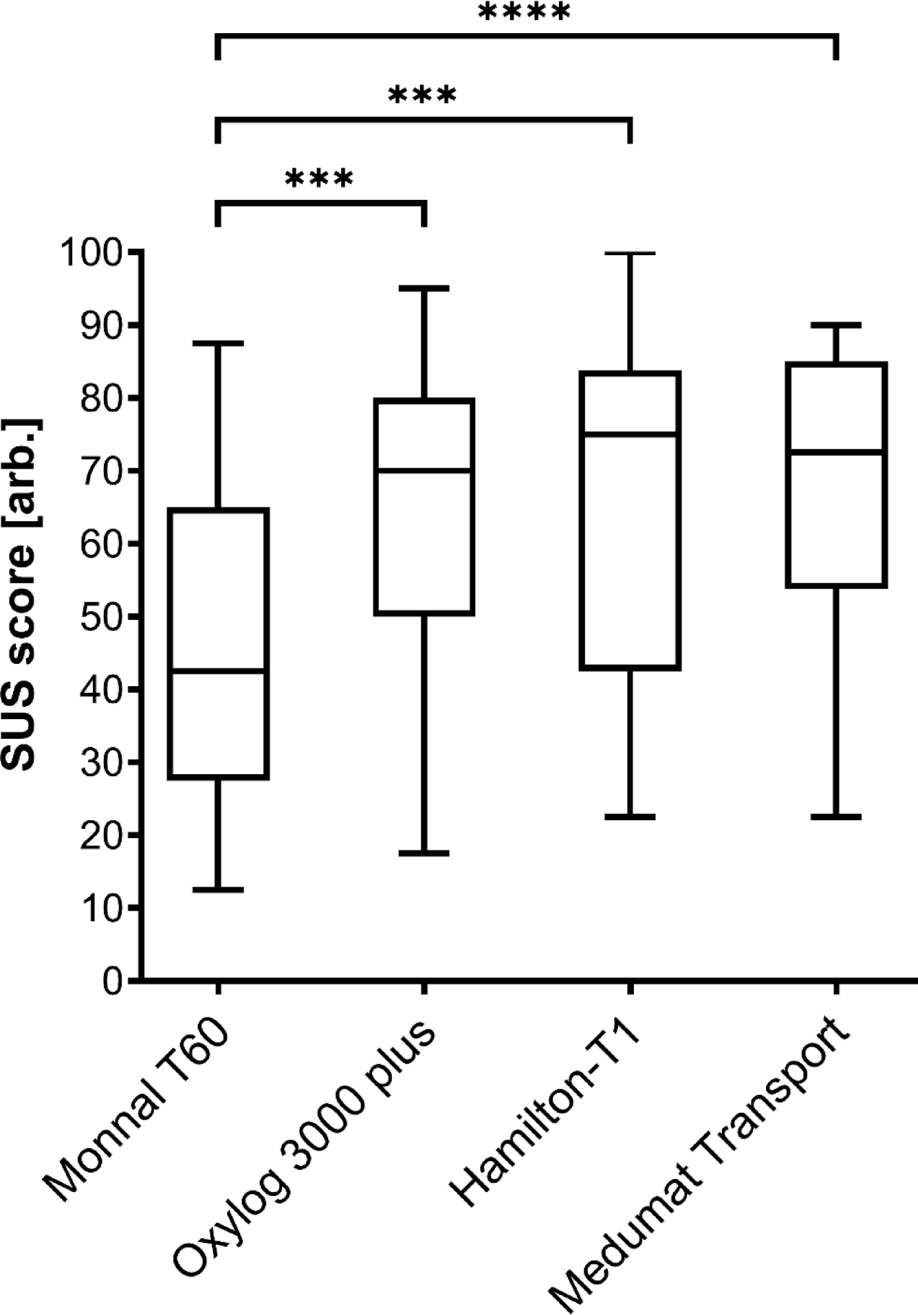
Scores according to the system usability scale (Brooke J 1996) (SUS) evaluated by the operators given for the four transport ventilators. ***P < 0.001, ****p < 0.0001.

SUS scores were comparable between the Oxylog 3000 plus, the Hamilton-T1 and the Medumat Transport, but significantly lower for the Monnal T60 (Fig. 3; *p* < 0.001). The perceived level of stress was higher after having used the Monnal T60 (43.1 [21.5]) compared to the Medumat Transport (29.5 [17.5]; *p* < 0.0001) and the Hamilton-T1 (31.4 [19.1]; *p* = 0.001) but did not differ significantly between the Oxylog 3000 plus (32.2 [21.8]) and all other devices (all *p* > 0.07). When asked about the comprehensibility of the feedback from the devices, operators rated the Hamilton-T1 best (3.0 [0.7]; *p* < 0.0001 vs. Monnal T60), followed by the Medumat Transport (2.6 [0.9]; *p* = 0.013 vs. Monnal T60), the Oxylog 3000 plus (2.2 [0.9]; n.s.) and the Monnal T60 (2.0 [0.8]; ANOVA *p* = 0.0004). When asked about the clarity of the screen display, the order of ratings was the same: Hamilton-T1 (2.9 [1.0]), Medumat Transport (2.5 [1.1]), Oxylog 3000 plus (1.8 [1.1]) and Monnal T60 (1.3 [1.2]; ANOVA *p* < 0.0001).

### Eye-tracking analyses

Hot spot measurements in the standby mode showed, that labelled items attracted visual attention (Supplement Fig. S1). Gazes were clearly directed towards the labelled keypads on the right side of the frontal screen of the Hamilton-T1. The most frequently visited AOIs in the other devices were the respective companies´ logos.

**Fig. 4:**
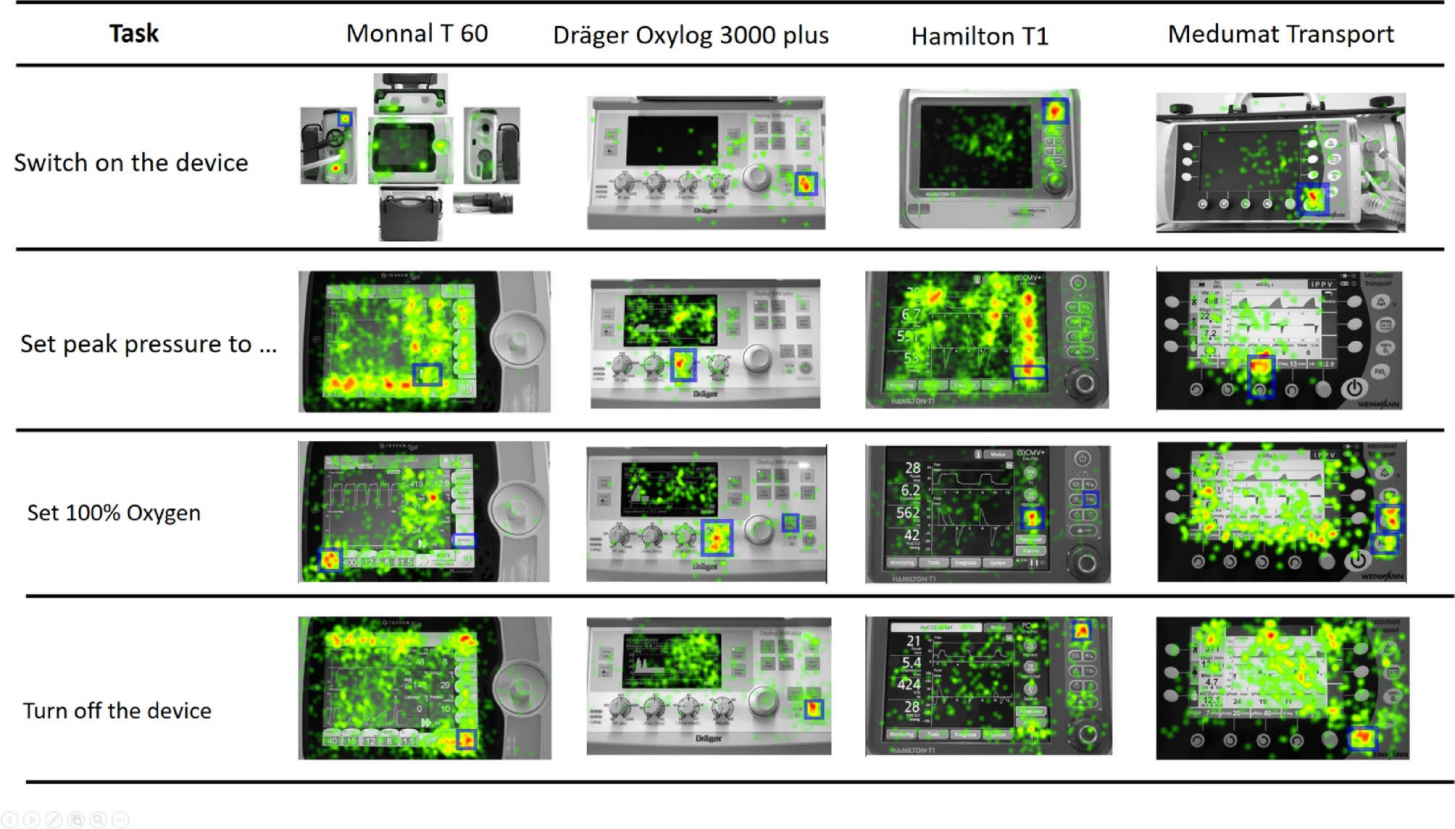
Heatmaps for selected tasks, where operators´ mean fixation count appear colored. Warm colors (red) represent a high number of fixations, followed by yellow and green. Blue squares represent the area of interest to indicate the screen area or button in question.

TFF varied depending on the task and device in question (Table 2). Heatmaps for selected tasks demonstrate the operators’ visual attention (Fig. 4).

**Table 2:** Time to first fixation (TFF) based on eye-tracking metrics for tasks showing significant differences in at least one comparison between devices.

Tasks	Monnal T60	Oxylog 3000 plus	Hamilton-T1	Medumat Transport
	[s]	[s]	[s]	[s]
Turn on the device	18.0 [14.4]	0.8 [0.4]	1.0 [0.8]	0.8 [0.4]
Set VCV mode	8.1 [5.0]	3.9 [2.8]	1.7 [1.9]	7.9 [7.6]
Set vent. Frequency to…	1.2 [1.0]	1.2 [1.0]	13.9 [13.0]	1.8 [0.9]
Set PEEP to…	1.5 [1.5]	9.8 [8.8]	1.8 [1.2]	1.6 [1.1]
Set peak pressure to…	43.5 [25.5]	2.3 [1.8]	44.3 [24.1]	1.5 [0.7]
Set I:E ratio to…	16.0 [17.2]	2.7 [2.4]	6.4 [2.9]	2.1 [0.9]
Read out P_peak_	7.2 [8.8]	19.0 [13.2]	3.4 [2.4]	12.2 [26.0]
Read out exp. MV	3.9 [2.6]	8.2 [4.7]	0.8 [0.8]	3.8 [3.1]
Set PSV mode	7.7 [1.8]	3.2 [1.8]	11.9 [10.7]	10.4 [6.2]
Open alarm menu	1.5 [2.8]	3.0 [1.7]	1.8 [1.1]	8.8 [4.6]
Set alarm volume to…	21.3 [12.3]	20.8 [19.4]	34.9 [25.8]	17.5 [11.7]
Read out end-tidal CO_2_	3.4 [5.4]	9.7 [1.8]	2.7 [0.8]	2.5 [1.6]
Read out exp. VT	12.1 [6.4]	10.6 [2.9]	1.5 [1.7]	3.6 [2.8]
Turn off the device	7.8 [12.6]	1.8 [2.2]	1.9 [1.1]	1.7 [0.9]

## Discussion

The main finding of the present study is that user acceptance of transport ventilators varies considerably between devices from different manufacturers. Overall user satisfaction was good for all devices except the Monnal T60. For this device, comparatively low ratings resulted from difficulties with the user interface, as confirmed by the operators’ comments and perceived stress levels.

By trend, subjectively experienced usability was supported by our objective measures. Systems with higher ratings were associated with fewer assistances and shorter task completion times. Gaze analysis based heatmaps revealed specific strengths and weaknesses of the devices’ interfaces.

### Connecting breathing hoses

The connection of the breathing hoses and associated sensors to the ventilator can be considered a critical issue for system failure leading to ventilator-related incidents ^12^. In the present study, each device required a specific breathing system. The Oxylog 3000 plus and Medumat Transport use a single tube system whereas the Monnal T60 and the Hamilton-T1 use a dual-tube system, leading back the expiratory air. With the latter, some operators were confused about the connection ends for the inspiratory and expiratory limb. Although the connection points are labelled at the device, the tubes are not properly labelled. Search for matching connection points resulted in a high error rate and long TCT in the Hamilton-T1. In addition, on the Hamilton-T1, the humidifier connector and the CO_2_-sample line are marked with the same color, leading some users to make incorrect assignments. Although most operators were able to do this without further assistance, the overall task completion time was delayed. Dealing with different solutions for the same task on different devices could have influenced users’ expectations as outlined by the DIN EN ISO 9241–110 standard (www.usability.de). If user experiences do not correspond to generally accepted conventions or familiar models, problems in interacting with the systems are to be expected.

### Power on

Switching on the device is obviously one of the most important and safety-relevant tasks. We found, however, significant long TCT and a diffuse search pattern while using the Monnal T60. In contrast to the other devices, the power button is located on the left site of the machine. However, our operators searched the front panel and mainly tried to activate the machine using the rotary knob, as this is the only hard key available on the Monnal´s front panel. Even those who inspected the left side paid more attention to the battery case than to the power button, as demonstrated by the respective heatmaps. Occasionally, the battery was even accidentally ejected thereby, resulting in a long overall TCT. Locating the power button on the left side may not have met the expectations of our users. In a study in ICU respirators, the majority of operators looked for the power button on the right site of the device, an observation the authors attributed to the prevalence of right-handed people ^13^.

Interestingly, at the end of the experiments, the operators also struggled to switch off the Monnal T60, which requires a sequence of several steps. A message on the touchscreen prompts the user to `press the button´ in order to continue, without further instructions. Most operators expected this to be the power button, but the rotary knob was requested instead. Asymmetrical design of operating steps can improve safe use as proposed by Vignaux et al. ^14^. However, learnability describes another principle for user-friendly interactive system according to ISO 9241–110, but proves to be insufficiently addressed in the process of activation and deactivation of this device.

### Set VCV

Setting of the ventilation mode and ventilation parameters demonstrates the impact that simple display structure and labelling can have on user satisfaction. The VCV ventilation mode is clearly labelled with a soft key on the Hamilton-T1 and a hard key on the Oxylog 3000 plus. In contrast, the quickest way to activate VCV in the Monnal T60 and Medumat Transport is to activate an emergency ventilation. The majority of the operators did not consider VCV to be a specific emergency ventilation mode and looked for an alternative way of activation. In the Medumat Transport, however, activation of VCV requires a number of consecutive steps, including specification of patient characteristics. This led to long TCT in this device.

### Set peak pressure

Limiting peak airway pressure represents an important function for patient safety. Most operators were able to quickly find the appropriate hard keys at the Oxylog 3000 plus and the Medumat Transport. Direct operation of the peak pressure is not available in the Hamilton-T1 and the Monnal T60. This can only be done indirectly, as specified in the instruction manual, by setting the upper limit of the airway pressure alarm in the Alarm Settings sub-menu. Making additional steps mandatory to set the value, operators needed considerably more time.

### Set fraction of inspired oxygen

In the critical event of oxygen desaturation, setting of inspired oxygen fraction is of crucial importance. Especially before tracheal suctioning or planned disconnection, the inspiratory oxygen concentration should be increased ^15^. Acute hypoxaemia also requires the immediate administration of an increased oxygen concentration. Users should therefore be able to change this setting without any delay. All devices provide a function that allow the user to directly increase the concentration of oxygen or even set it directly to 100%. However, it was only on the Medumat Transport that the majority of operators made use of this function. Gaze analysis revealed that clear labelling and positioning of the hardkey next to the FIO_2_ function may have promoted the users’ action in this device.

Analysis of eye-tracking heatmaps showed that positioning and visibility (e.g. the same shape and size as the FiO_2_ function), clear labelling and accessibility without intermediate steps can help function keys to be recognized as efficient options. This result suggests that the most important functions for acute ventilation therapy should be made available as hard keys.

### Set pressure support ventilation mode

It is noteworthy that ventilation modes are not consistently named by the manufacturers represented in our study. The supported spontaneous ventilation mode is an example of this. Although all operators were familiar with the technical possibilities of supporting spontaneous breathing during mechanical ventilation, the activation of pressure supported ventilation led to the highest failure rate per task, accounting for approximately 42% of all errors. In the Hamilton-T1, this mode is labelled `SPONT´, which does not allow any conclusion to be drawn about the type of pressure support provided. In the Oxylog 3000 plus and Medumat Transport, inspiratory pressure support is available in augmented continuous positive airway pressure modes, labelled `Spont-CPAP´ and `CPAP + ASB´ respectively, which most of our operators did not identify as such function. Interestingly, although the Monnal T60 was the only device to offer PSV clearly labelled as suggested by the above ISO Standards, most users chose CPAP instead. As no feedback was provided on the success of the tasks performed, false experience with the other devices may have led some operators to follow this approach. Different terminology is considered to be a relevant issue recently addressed by the international ISO committee ^16^. A key factor affecting the usability of ventilators could be the lack of a standardized vocabulary for how these devices work ^17^. Accordingly, training on ventilator terminology should be enforced, as suggested by a small pilot study ^18^ and the self-descriptiveness of labeling should be considered very important in accordance with the principles for user-friendly systems.

A selected collection of measures for potential usability improvement from these analyses is given in Table 3.

**Table 3:** Selection of measures for improving usability.

Measure	Effect on usability	Example
Complete labeling and identification of items	Visual attention, direct access	Labelling of inspiratory and expiratory connection at device and tubing system
Clear labeling/clear user guidance	Reduced confusion	‘Press rotary knob’ instead of ‘Press button’
Direct access to important functionality	Fast approach to tasks solving	Hard keys for setting peak pressure instead peak pressure set via upper limit of the respective alarm
Consistent labeling	Facilitated training, reduced cognitive workload	PSV instead SPONT/CPAP + ASB
Meet expectation/convention	General intuitivity	On-switch on the right side instead on left side
Decent logo	Reduced distraction	

## Limitations of the study

We chose a calm simulation environment for our experiments in order to allow for a differentiated analysis of machine related operating problems. However, a real-world contextual setting would increase the cognitive load on the operator, for example through movement and difficult access to the ventilator interface. Moreover, the operator would have to share attention with additional parameters such as vital parameter monitoring and medication. As a consequence, it can be assumed that processing times would have been longer under more realistic test conditions. Moreover, in the real-life application of the transport ventilators it will not be possible to obtain immediate assistance to compensate for shortcomings in usability. Accordingly, task failure rates would likely be increased. Therefore, user interface deficiencies found in our study may become more relevant in the context of the intended use of the devices, which needs to be clarified in further research.

We selected relatively inexperienced staff for our study. Training may partially compensate for the devices’ shortcomings identified in our study. However, benefits resulting from training rely on memory, which may not always be accurate or complete. Therefore, manufacturers should always be encouraged to improve fault tolerance by human factors engineering where possible ^5^.

The experience of some operators with the Dräger Oxylog and Dräger anesthesia machines, may have influenced comparability of the results. With respect to the relevance of the Dräger Oxylog in the European market, however, we decided to include this device regardless of the users’ experiences. Usability tests are best conducted by individuals who are familiar with the context of use, taking into account a heterogeneous level of experience. A previous study demonstrated that even users who are familiar with a device can have an high error rate ^2^.

Presentation of the tasks in fixed instead of randomized order may have influenced our results due to the fact that this also includes a fixed course of exploration of the respective device during the task series. However, as stated earlier, randomized task presentation may have resulted in conflicting order of tasks. For example, ‘Read out minute volume’ before ‘Start ventilation’ would have required an interruption of the experiment for an intervention of the investigator, thereby the operator had to be prevented from observing the intervention. Thus, randomization of the tasks would not have been be feasible without significantly limiting the selection of the examined tasks.

In this context, we cannot rule out that working through the menu structure for a certain task potentially helped navigating the menu structure for a later task. This could affect the perceived difficulty of each task differently for the various ventilators, depending on how much can be learned from the first tasks with each device.

## Conclusion

Our study identified task-specific shortcomings in the usability of representative transport ventilators revealing non-intuitive layout as potential source for delay or even fail of specific tasks processing. Clear and consistent labelling as well as the provision of hard keys for functions relevant to patient safety may reduce the risk of human factor related errors. Therefore we recommend that manufacturers take task-specific shortcomings into account in their medical device instructions and device development.

## Key messages

First, our results show that the task-specific usability depends on the design of the respective item of the user interface.

Second, gaze analysis of the four devices reveals non-intuitive layout as a potential sources of error.

Third, our results suggest that the optimal user interface of a transport ventilator may include both touch screen and hard keys. Functions of fundamental importance for patient safety, such as starting the device, setting alarm limits and inspiratory concentration of oxygen, should be provided as clearly labelled hard keys. Moreover, clear labelling of all connection points on the device and related equipment may avoid time delay during assembly and, more importantly, incorrect connections.

Fourth, our results demonstrate that there is a need for uniform labelling of common functions beyond ISO standards ^16^ to avoid distraction and delay in the operation of the devices.

For the time being, users and device trainers may use the provided data to draw more attention to the specific weaknesses of the devices, thus increasing patient safety from the provider’s side.

## Supplementary Information

Below is the link to the electronic supplementary material.

Supplementary Material 1

## Data Availability

The datasets generated and analysed during the current study are not publicly available due to a large dataset but are available from the corresponding author on reasonable request.

## Abbreviations

AOI: Area(s) of interest
ASB: Assisted spontaneous breathing
CPAP: Continuous positive airway pressure
FDA: U.S. Food and Drug Administration
ICU: Intensive care unit
ISO: International Organization for Standardization
MV: Minute ventilation
PEEP: Positive end-expiratory pressure
PSV: Pressure support ventilation
SD: Standard deviation
SPONT: Spontaneous breathing
SUS: System usability scale
TCT: Task completion time
TFF: Time to first fixation
VCV: Volume controlled ventilation
VT: Tidal volume

## Declarations

### Ethial approval

Approval of the Ethics Committee of the University of Freiburg, Germany (21.02.2017, Approval Number 56/17). Each operator gave written informed consent before inclusion in the study.
