# Supplementary material for: Qualitative and quantitative evaluation of the usability of transport ventilators using eye tracking
Source: Sci Rep. 2026 Jan 8;16:886. doi: 10.1038/s41598-025-34154-5 (PMC12783852; doi:10.1038/s41598-025-34154-5)
Supplement: Supplementary file 1 — Supplementary Material 1 [file 41598_2025_34154_MOESM1_ESM.docx]

**Supplement Figure_S1 Hot Spots**


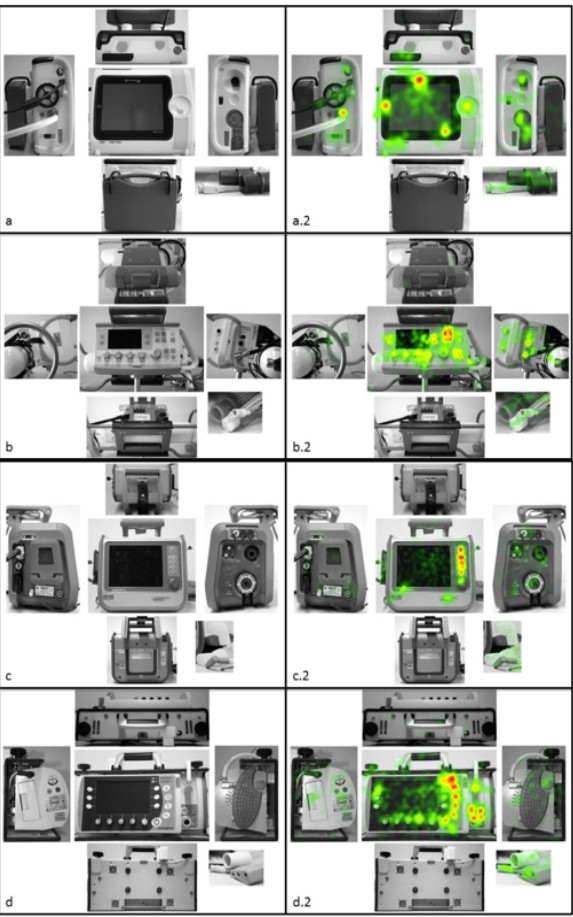


**Supplement Figure_S1 Hot Spots:** All-round view of the devices and hotspots: Distribution of the first 50 fixation points of all operators on the user interface of the respective device. Colour-coded: red areas represent a high number of fixations. a Monnal T60, b: Oxylog 3000 plus, c: HAMILTON-T1, d: Medumat Transport
